# The enigma of mixed connective tissue disease—challenges in routine care

**DOI:** 10.1007/s10067-022-06286-w

**Published:** 2022-07-29

**Authors:** Adrian Wanzenried, Alexandru Garaiman, Suzana Jordan, Oliver Distler, Britta Maurer

**Affiliations:** 1 Department of Rheumatology, University Hospital Zurich, University Zurich, Zurich, Switzerland; 2 Department of Rheumatology and Immunology, University Hospital Bern, University Bern, Bern, Switzerland

**Keywords:** Diagnosis, Disease activity, Mixed connective tissue disease, Treatment

## Abstract

**Objectives:**

As a rare and heterogeneous disease, mixed connective tissue disease (MCTD) represents a challenge. Herein, we aimed to unravel potential pitfalls including correct referral diagnosis, distinction from other connective tissue diseases (CTD) and treatment modalities.

**Methods:**

We characterised the MCTD cohort at our tertiary referral centre. All patients were evaluated for fulfilment of classification criteria of various CTDs. SLEDAI-2 K and EUSTAR-AI were used in accordance with previous research to evaluate disease activity and treatment response.

**Results:**

Out of 85 patients initially referred as MCTD, only one-third (33/85, 39%) fulfilled the diagnostic MCTD criteria and the other patients had undifferentiated CTD (16/85, 19%), non-MCTD overlap syndromes (11/85, 13%) and other rheumatic diseases. In our final cohort of 33 MCTD patients, 16 (48%) also met the diagnostic criteria of systemic sclerosis, 13 (39%) these of systemic lupus erythematosus, 6 (18%) these of rheumatoid arthritis and 3 (9%) these of primary myositis. Management of MCTD required immunomodulating combination therapy in most cases (15/28, 54%), whereas monotherapy was less frequent (10/28, 36%), and only a few (3/28, 11%) remained without immune modulators until the end of the follow-up period. Treatment led to a significant decline in disease activity.

**Conclusions:**

Our study showed a high risk for misdiagnosis for patients with MCTD. As a multi-organ disease, MCTD required prolonged immunomodulating therapy to achieve remission. The establishment of an international registry with longitudinal data from observational multi-centre cohorts might represent a first step to address the many unmet needs of MCTD.

**Table:** 

**Key Points** *• This cohort study aimed to identify challenges in the highly complex management of MCTD.* *• Clinical presentation of MCTD significantly overlaps with that of other CTDs, leading to a high risk of misdiagnosis.* *• Manifestations of MCTD are highly variable and potentially life-threatening, requiring continued immunomodulating treatment in most cases.* *• A composite score based on SLEDAI-2 K and EUSTAR-AI measures could represent an easy applicable tool to monitor disease activity and treatment response.*	

## Introduction

Mixed connective tissue disease (MCTD) is a systemic, immune-mediated disorder exhibiting a broad spectrum of disease manifestations including symptoms of systemic sclerosis (SSc), systemic lupus erythematosus (SLE), primary myositis (PM) or rheumatoid arthritis (RA), which underline the importance of high titres of anti-U1-snRNP autoantibodies for diagnosis. MCTD was first described in 1972 by G. C. Sharp and colleagues [1]. Since then, there has been an ongoing debate about whether it represents a distinct disease entity [2, 3] or rather an early stage of another connective tissue disease (CTD) or an unspecific overlap syndrome [4]. Evidence for MCTD as a distinct disorder has mainly come from research into genetics and immunology: HLA-typing studies identified characteristic risk alleles for MCTD compared to other CTDs [5] or healthy controls [6]. Immunologic studies have elucidated the role of anti-U1-snRNP autoantibodies in the pathogenesis of MCTD [7, 8].

Challenges in dealing with MCTD arise for many reasons. Firstly, MCTD is the most rare of all CTDs with a prevalence of 3.8/100,000 adults in a Norwegian study [9]. Secondly, in the absence of specific guidelines for MCTD, treatment is based on personal expertise from treating similar symptoms in other CTDs [2]. Thirdly, four different diagnostic and classification criteria with varying defining features and sensitivities have been proposed [10–13]. This does not only cause diagnostic uncertainty in clinical routine, but has also led to heterogeneous study cohorts in previous MCTD research endeavours [14]. Finally, some patients even fulfil classification criteria of various other CTDs due to the significant overlap between the symptoms of MCTD and SSc, SLE, PM or RA [2].

In this study, in our tertiary referral centre, we aimed to unravel potential pitfalls of the overall management of MCTD, including correct referral diagnosis, fulfilment of diagnostic and/or classification criteria, distinction from other CTDs and disease course and activity as well as treatment modalities.

## Materials and methods

### Study cohort

This is a retrospective study performed at the Department of Rheumatology, University Hospital Zurich, a tertiary referral centre. We searched all electronic medical reports of our department from January 2015 to December 2018 for patients diagnosed with M35.1, the code assigned to MCTD in the 10th Revision of the International Classification of Diseases (ICD-10). The search yielded 85 patients. In addition, our local cohort of SSc patients, whose data is recorded in the European Scleroderma Trials and Research (EUSTAR) database [15], was evaluated for patients fulfilling MCTD criteria. At the time of the data export from the EUSTAR database (26 July 2019), the data set of our centre consisted of 495 patients, of which 15 patients were positive for anti-U1-snRNP. The inclusion criteria used for patient selection were age ≥ 18 years, fulfilment of diagnostic MCTD criteria (either Sharp’s [10], Kasukawa’s [11], Alarcón-Segovia’s [12] or Kahn’s criteria [13]) and anti-U1-snRNP antibody titre > 100 U/ml. All participants provided informed consent and the Zurich Ethics Committee approved the study (approval number: 2020–00387).

### Diagnostic and classification criteria

**Fig. 1:**
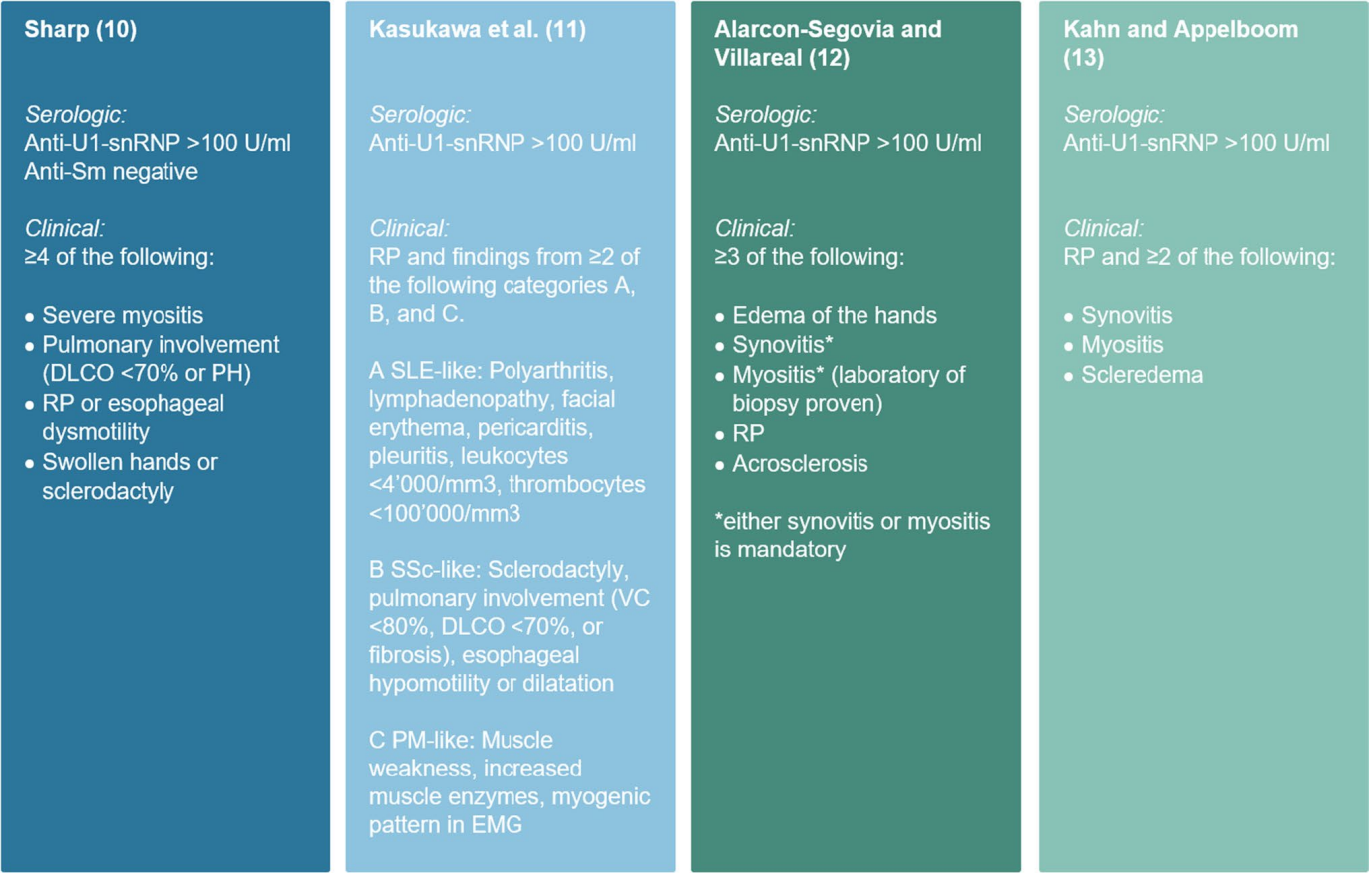
Diagnostic criteria for definite MCTD. The criteria have been slightly modified for use in this study as described in the text. Anti-Sm, anti-Smith; DLCO, diffusion capacity for carbon monoxide; EMG, electromyography; PH, pulmonary hypertension; RP, Raynaud’s phenomenon; VC, vital capacity

The diagnostic MCTD criteria were applied in accordance with the original publications. However, we had to adjust for the definition of the autoantibody thresholds. Since anti-U1-snRNP is measured in units per millilitre (U/ml) at our department, we regarded a titre of > 100 U/ml as sufficient instead of the anti-RNP hemagglutination titres of > 1:1600 in Alarcón-Segovia’s criteria [12], > 1:2000 in Kahn’s criteria [13] and instead of the anti-ENA titre of > 1:10,000 in Sharp’s criteria [10]. The minimum anti-U1-snRNP threshold of > 100 U/ml was chosen since this reflects exceedingly high titres based on our internal reference values (upper limit of normal = 25 U/ml). The diagnostic criteria for definite MCTD as used in this study are displayed in Fig. 1. For classification, we used the SLICC criteria for SLE [16], the ACR/EULAR criteria for SSc [17], the ACR/EULAR criteria for PM [18] and the ACR/EULAR criteria for RA [19]. Fulfilment of all mentioned classification criteria was evaluated for each patient of our local MCTD cohort.

### Clinical parameters

Patients were assessed at their first visit to our department after referral. Clinical data were retrospectively collected by manual review of the electronic patients’ records. The collected data comprise a detailed patient’s history, detailed evaluation of clinical features and additional diagnostic exams. Results of the following diagnostic procedures were recorded: routine blood and urine testing, auto-antibody screening, nailfold capillaroscopy, pulmonary function tests, high resolution computed tomography of the chest (HRCT), conventional chest radiography, electrocardiography, echocardiography, right heart catheter examination, electromyography (EMG), whole body magnetic resonance tomography (MRI) and muscle biopsy. Nailfold capillaroscopy was evaluated for the presence of microangiopathy according to the guidelines of Smith et al. [20]. Myositis was defined as muscle symptoms associated with elevated muscle enzymes or pathologic findings on MRI or EMG.

### Immunomodulatory treatment and disease activity

We also collected longitudinal data to assess for disease course, immunomodulating treatment, treatment response and drug-related adverse events (AE). All documented visits to our department from the first visit after referral to either time of data collection or loss of follow-up were manually reviewed. Patients with an observation time < 1 year were excluded from this part of the analysis.

We relied on validated disease activity measures for the assessment of disease course and treatment response. As proposed by Reiseter et al*.* [14], we used SLEDAI-2 K as defined by Gladman et al*.* [21] and EUSTAR Activity Index as defined by Valentini et al*.* [20], which are validated for SLE and SSc, respectively, to measure disease activity in our MCTD cohort. Immunomodulating drugs encompassed corticosteroids, antimalarials, methotrexate, sulfasalazine, azathioprine, mycophenolate mofetil, cyclophosphamide, cyclosporine A, TNFα-antagonists, rituximab and tocilizumab.

### Analysis of data

Absolute and relative frequencies were calculated for nominal variables; median and interquartile range was calculated for continuous variables. Disease activity was evaluated for the first and last visit at our department based on EUSTAR-AI [21] and SLEDAI-2 K [22]. Use of immunomodulating drugs and occurrence of AEs were continually recorded through the follow-up period. Data analysis was performed on Microsoft Excel and R 4.0 [23].

## Results

### Identification of MCTD patients

**Fig. 2:**
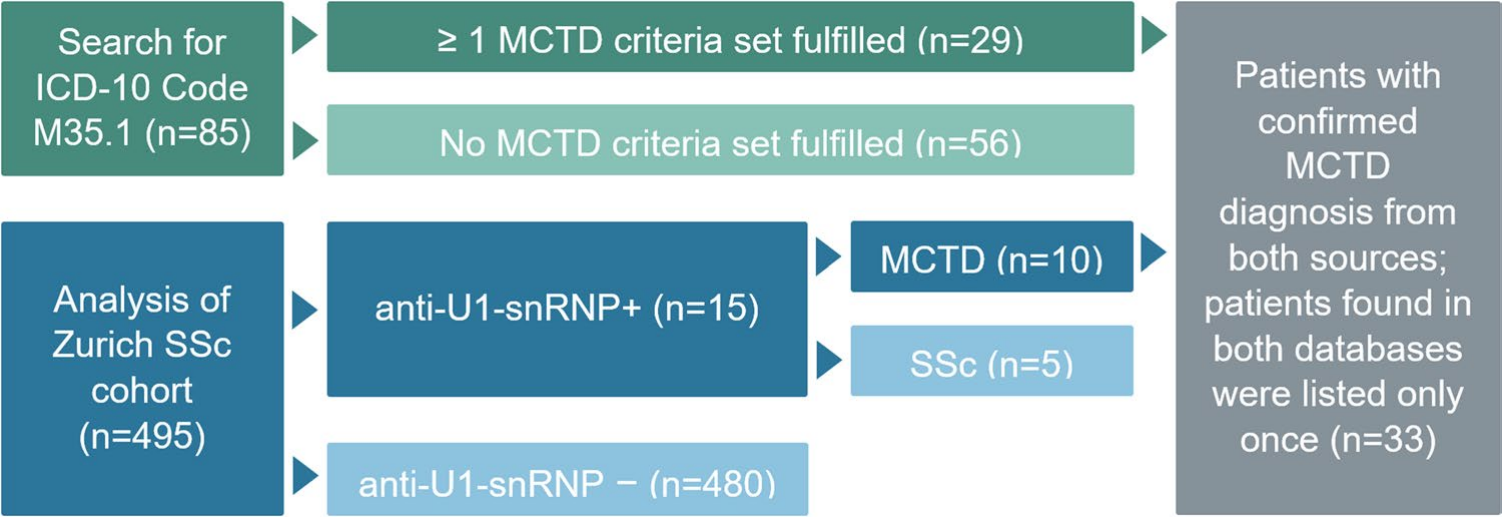
Search strategy for identifying MCTD patients in our department

The selection process of the study cohort (*n* = 33) is shown in Fig. 2. The search for patients with the ICD-10 Code M35.1, which depicts MCTD, yielded 85 patients. On detailed review by two independent reviewers (AW, BM), 29/85 (34%) patients fulfilled at least one of the four MCTD criteria sets (Sharp’s [10], Kasukawa’s [11], Alarcón-Segovia’s [12] or Kahn’s [13] criteria), whereas 56/85 (66%) patients did not fulfil any MCTD criteria set. From the Zurich SSc cohort (*n* = 495) at the time of the data export (26 July 2019), 15 patients were positive for anti-U1-snRNP. On detailed review (AW, BM), 10/15 patients not only were anti-U1-snRNP positive, but also exhibited findings inconsistent with SSc diagnosis and instead rather fulfilled MCTD criteria. Thus, our final MCTD dataset yielded 33 MCTD patients and comprised patients identified by search for diagnostic code alone (*n* = 23), by search for autoantibody alone (*n* = 4) or by both methods (*n* = 6).

### Baseline characteristics

The patients’ demographic and clinical characteristics at first visit to our department are shown in Table 1. Most patients of our cohort were female with a median age of 53 years. Main clinical characteristics included Raynaud’s phenomenon (100%), puffy fingers (64%), scleroderma pattern in nailfold capillaroscopy (57%), oesophageal symptoms (55%), synovitis (55%), positive rheumatoid factor (45%), hypocomplementemia (42%), lung fibrosis on HRCT (39%), dyspnea (37%), sclerodactyly (36%), myositis (33%), muscle weakness (30%), CK elevation (30%) and joint contractures (29%). Median FVC was 92%; median DLCO was 67%.

**Table 1:** Description of the study cohort at baseline (total *n* = 33)

Item	Absolute freq. or median	Relative freq. or interquartile range
Age (years)	53	_(38,59)_
Female	29/33	(88)
Male	4/33	(12)
Raynaud’s phenomenon	33/33	(100)
Dyspnea	11/30	(37)
NYHA I	19/30	(63)
NYHA II	7/30	(23)
NYHA III	4/30	(13)
NYHA IV	0/30	(0)
Oesophageal symptoms	18/33	(55)
Stomach symptoms	5/33	(15)
Intestinal symptoms	6/33	(18)
Puffy fingers	21/33	(64)
Sclerodactyly	12/33	(36)
Pitting scars	5/33	(15)
Digital ulcers	0/33	(0)
History of digital ulcers	2/33	(6)
Gangrene	0/33	(0)
Mechanic’s hands	0/33	(0)
Modified Rodnan skin score	0	_(0,4.25)_
Synovitis^1^	18/33	(55)
Joint contractures	8/28	(29)
Tendon friction rubs	5/25	(20)
Muscle weakness	10/33	(30)
Myositis	11/33	(33)
FVC (%)	92	_(78,100)_
TLC (%)	89	_(78,100)_
DLCO/SB (%)	67	_(56,74)_
Lung fibrosis on HRCT	13/33	(39)
Conduction blocks	3/26	(12)
LVEF (%)	64	_(62,66)_
Diastolic dysfunction	1/32	(3)
Pulmonary hypertension by Echo	4/33	(12)
Scleroderma pattern in NVC^2^	17/30	(57)
ANA	33/33	(100)
ACA	1/31	(3)
Anti-Scl70	0/31	(0)
Anti-U1-snRNP	33/33	(100)
Anti-RNA-polymerase III	1/26	(4)
Anti-PM-Scl	1/22	(5)
Anti-Jo-1	0/23	(0)
Rheumatoid factor	15/33	(45)
ESR (mm/1 h)	25	_(18,34)_
CRP elevation	4/32	(13)
CK elevation	10/33	(30)
Proteinuria	3/30	(10)
Serum creatinine (mg/dL)	0.73	_(0.64,0.80)_
Hypocomplementemia	14/33	(42)

The absolute and relative frequencies are shown for nominal variables: n/available (p%). For continuous variables, median and 1st and 3rd quartile are given: median (Q1, Q3). ^1^Synovitis was defined as swelling of a joint as judged by the treating physician. ^2^Scleroderma pattern as defined by Smith et al. (21). *ACA*, anti-centromere antibodies; *ANA*, antinuclear antibodies; *CK*, creatinine kinase; *CRP*, C reactive protein; *DLCO/SB*, single breath diffusion capacity of the lung for carbon monoxide; *ESR*, erythrocyte sedimentation rate; freq., frequency; *FVC*, forced vital capacity; *HRCT*, high-resolution computed tomography; *LVEF*, left ventricular ejection fraction; *NVC*, nailfold capillaroscopy; *NYHA*, New York Heart Association; *TLC*, total lung capacity

### Diagnostic overlap between MCTD and other CTDs, performance of diagnostic criteria

Challenges arise from the fact that MCTD patients often also meet the classification criteria of other rheumatic diseases including SSc [17], SLE [16], PM [18] and RA [19]. Indeed, most patients (25/33, 76%) fulfilled the classification criteria of at least one of the mentioned diseases in addition to MCTD. The SSc (16/33 patients, 48%) and SLE criteria (13/33 patients, 39%) were the most frequently fulfilled in our MCTD cohort.

The use of four different diagnostic MCTD criteria may further contribute to some diagnostic uncertainty in clinical routine. Performance of MCTD criteria showed the highest sensitivities for Kasukawa’s (31/33, 94%) and Alarcón-Segovia’s criteria (30/33, 91%). Notably, most patients (32/33, 97%) fulfilled more than one set of MCTD criteria. Fulfilment of MCTD, SSc, SLE, PM and RA diagnostic and classification criteria is summarised in Table 2.

**Table 2:** Patients fulfilling classification criteria of MCTD and other CTDs (total *n* = 33)

Diagnostic/classification criteria	Frequency
Sharp MCTD criteria [10]	25 (76%)
Kasukawa et al*.* MCTD criteria [11]	31 (94%)
Alarcón-Segovia and Villareal MCTD criteria [12]	30 (91%)
Kahn and Appelboom MCTD criteria [13]	28 (85%)
ACR/EULAR systemic sclerosis criteria [17]	16 (48%)
SLICC systemic lupus erythematosus criteria [16]	13 (39%)
ACR/EULAR rheumatoid arthritis criteria [19]	6 (18%)
ACR/EULAR primary myositis criteria [18]	3 (9%)
Patients fulfilling > 1 MCTD criteria set	32 (97%)
Patients fulfilling all 4 MCTD criteria sets	20 (61%)
Patients fulfilling MCTD and ≥ 1 other CTD criteria	25 (76%)
Patients fulfilling MCTD and ≥ 2 other CTD criteria	11 (33%)

The absolute and relative frequencies are shown: *n* (%)

### High risk of misdiagnosis in clinical practice

The challenge of MCTD diagnosis was further reflected in the high percentage of patients (56/85, 66%) referred as MCTD patients, but not fulfilling any MCTD criteria set. The corrected diagnoses of these patients are shown in Table 3. Undifferentiated CTD (16/56, 29%; [24]), non-MCTD overlap syndromes (11/56, 20%; [26]) and SLE (6/56, 11%; [16]) were the most frequent diagnoses mistaken for MCTD. However, numerous other rheumatic diseases including Sjögren’s syndrome, SSc and RA had also been mistaken for MCTD.

**Table 3:** Diagnoses of patients with ICD10 code M35.1 not fulfilling MCTD criteria (*n* = 56)

Corrected diagnosis	Frequency
Undifferentiated connective tissue disease [24]	16 (29%)
Non-MCTD overlap syndromes^1^	11 (20%)
Other^2^	10 (18%)
Systemic lupus erythematosus [16]	6 (11%)
Primary Sjögren’s syndrome [25]	5 (9%)
Unclassifiable connective tissue disease	4 (7%)
Systemic sclerosis [17]	2 (4%)
Rheumatoid arthritis [19]	2 (4%)

Absolute and relative frequencies are shown: *n* (%). ^1^Overlap syndromes include conditions fulfilling both serological and clinical criteria of two classic CTDs as described by Pepmueller (26). ^2^Other diagnoses include generalised morphea with concomitant polyarthritis, unclassified systemic inflammatory syndrome, systemic inflammation of unknown aetiology, autoimmune dacryoadenitis, chronic arthralgias with sicca syndrome, seronegative primary Sjögren’s syndrome, mechanically induced arthralgia of finger joints, somatoform pain disorder, human immunodeficiency virus infection and spondyloarthritis

### Prescription of immunomodulators, disease course and adverse events

Observation time from the first visit after referral to the time of data collection or loss of follow-up was ≥ 1 year in 28/33 (85%) patients. Longitudinal data of this subcohort mirrors the management of MCTD at our department including use of immunomodulating drugs, treatment response and adverse events (AE). The median follow-up time was 7.6 (2.9, 10.3) years.

In the absence of treatment guidelines for MCTD, treatment decisions are mainly driven by the clinically dominant features and are thus often modelled to the existing recommendations for SSc [27, 28], SLE [29], PM [30] or RA [31]. We therefore examined the prescription patterns in our MCTD patients followed over ≥ 1 year (*n* = 28/33): Hydroxychloroquine, prednisone, methotrexate and rituximab were prescribed to 23/28 (82%), 22/28 (79%), 20/28 (71%) and 10/28 (36%) patients over the follow-up period. Mycophenolate mofetil, tocilizumab, azathioprine, leflunomide, cyclophosphamide, TNF-α-antagonists, sulfasalazine and cyclosporine A were less frequent treatment choices. The most frequent indications for treatment initiation were arthritis/arthralgia and interstitial lung disease.

**Fig. 3:**
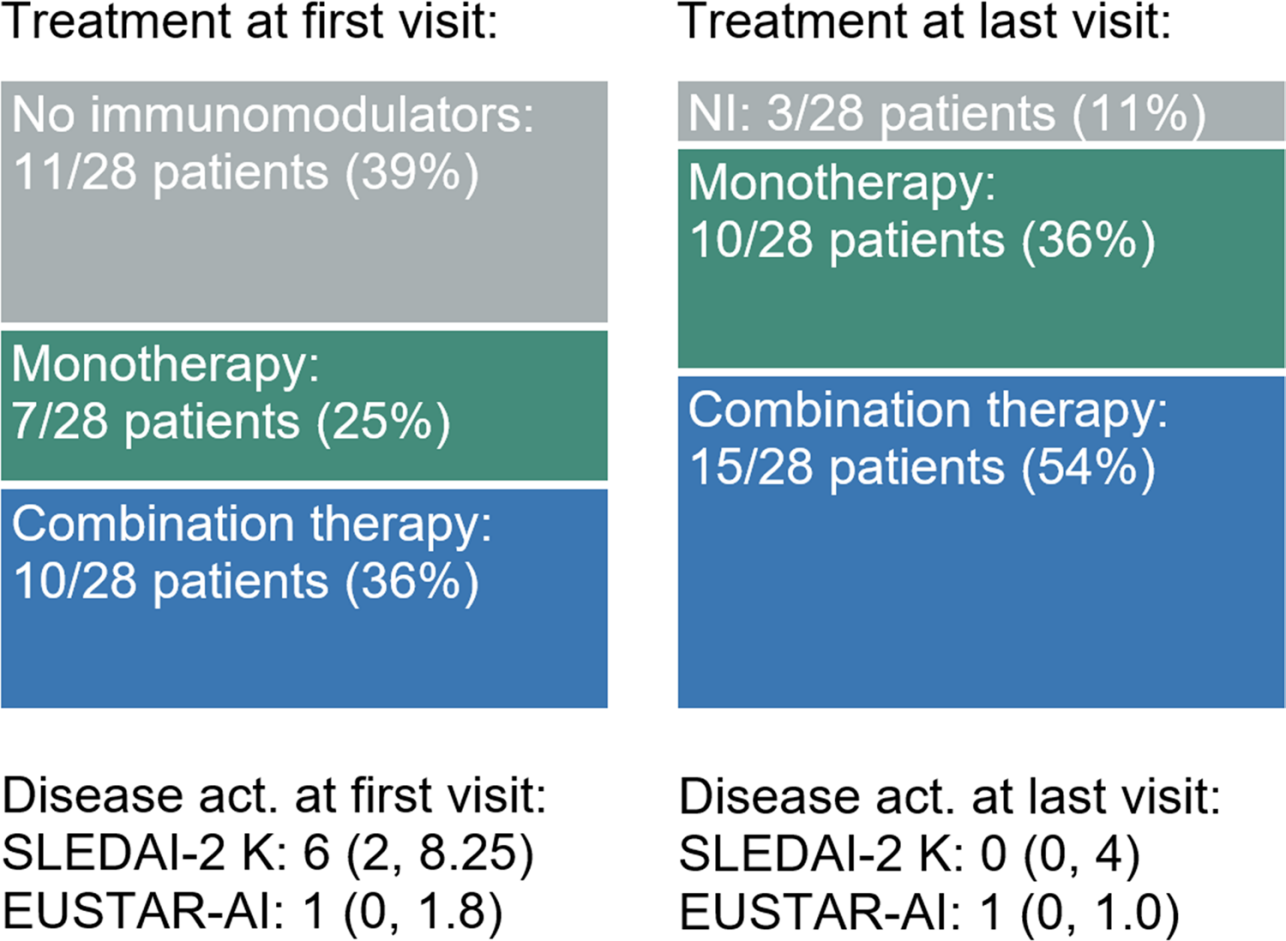
Prescription patterns and disease activity at first and last (time of data collection or loss of follow-up) visit to our department. Median, 1st and 3rd quartile of the disease activity measures SLEDAI-2 K [22] and EUSTAR-AI [21] are displayed as median (Q1, Q3). Act, activity; NI, no immunomodulators

Having evaluated the preferentially prescribed immunomodulators, we next examined whether these agents were used as monotherapy or in combination. Combination therapy was favoured over monotherapy in most patients with further increase over the follow-up period, probably reflecting the severity of the multi-organ disease. At the time of data collection or loss of follow-up, 15/28 (53%) patients were on combination therapy, 10/28 (36%) on monotherapy and just 3/28 (11%) off therapy as is shown in Fig. 3.

In agreement with previous research [14], we used SLEDAI-2 K as defined by Gladman et al. [21] and EUSTAR Activity Index as defined by Valentini et al. [20], which are validated for SLE and SSc, respectively, to measure disease activity in our MCTD cohort in order to assess the disease course and treatment response in our MCTD patients. Median (Q1, Q3) SLEDAI-2 K significantly decreased from 6 (2, 8.25) at the first visit to our department to 0 (0, 4) at the time of data collection or loss of follow-up, whereas median (Q1, Q3) EUSTAR-AI hardly changed, being 1 (0, 1.8) at the first visit to our department and 1 (0, 1.0) at the time of data collection or loss of follow-up. The development of immunomodulator prescription patterns and disease activity is summarised in Fig. 3.

Finally, we assessed the frequency and nature of adverse events (AEs) in our cohort. Throughout the follow-up period, 17/28 (61%) patients experienced at least one AE, and 9/28 (32%) experienced more than one AE. There were AEs associated with methotrexate in 12/28 (43%) patients, with hydroxychloroquine in 7/28 (25%) patients, and with the concurrent use of several immune-modulating drugs in 4/28 (14%) patients. Cytopenia (8/28 patients, 29%), exanthema (6/28 patients, 21%), infections (4/28 patients, 14%) and elevated liver enzymes (4/28 patients, 14%) were the most frequently recorded AEs in our cohort.

## Discussion

**Fig. 4:**
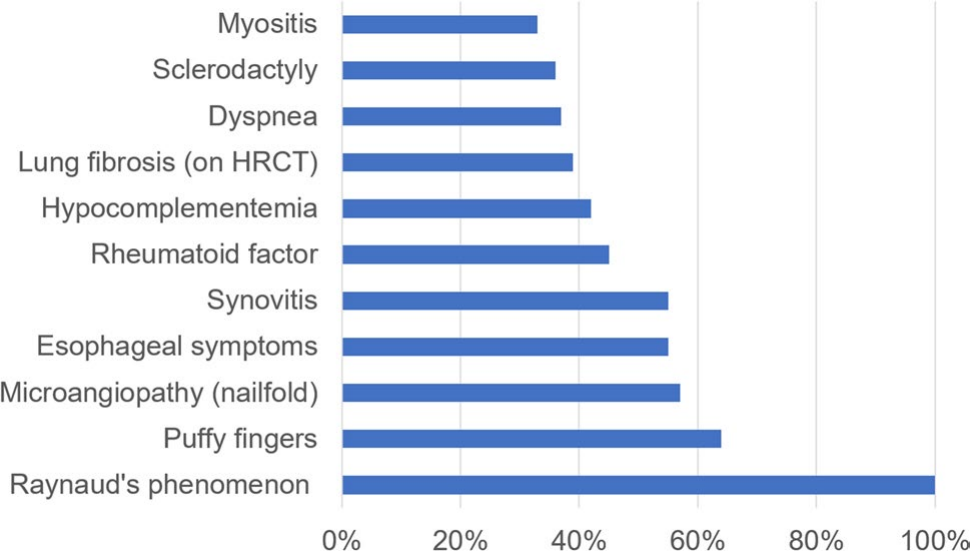
Most frequent clinical findings in our MCTD cohort (*n* = 33)

Our MCTD dataset comprised mainly females in their fifth or sixth decade with multi-organ involvement, particularly peripheral microangiopathy, puffy fingers, sclerodactyly, dysphagia, arthritis, interstitial lung disease and myositis (Fig. 4). A nationwide Norwegian cohort study [14] described similar frequencies of the mentioned disease manifestations, except in arthritis which occurred somewhat less frequently in our study (55% in our cohort, 76% in the Norwegian cohort).

Since these features are not pathognomonic for MCTD, most of our patients (25/33, 76%) fulfilled not only the MCTD criteria, but also the classification criteria for SSc, SLE, RA or/and PM. This is consistent with the previously proposed view of MCTD as an overlap syndrome that is defined by its association with anti-U1-snRNP [26]. Previous studies have also described simultaneous fulfilment of MCTD and other CTD criteria [2, 32, 33].

The respective choice of the diagnostic MCTD criteria set might also influence the establishment of the diagnosis. In our cohort, 97% of the patients fulfilled several criteria sets. Sensitivity was highest for Kasukawa’s (94%; [11]), followed by 91% for Alarcón-Segovia’s [12], 85% for Kahn’s [13] and 76% for Sharp’s [10] criteria. Previous studies also reported significant differences in the performance of MCTD criteria [9, 34].

As a disease with a broad spectrum of clinical manifestations, significant overlap with other rheumatic diseases, heterogeneous use of diagnostic criteria and low disease prevalence, MCTD represents a significant diagnostic challenge even for rheumatologists. In our study, the majority (66%) of referred “MCTD” patients did not fulfil MCTD criteria but rather had other conditions including undifferentiated CTD as defined by Mosca et al. [24] and non-MCTD overlap syndromes (meeting clinical and serological criteria of more than one classic CTD [26]).

Clinical decision-making in the absence of official recommendations and randomised controlled trials might be difficult [2, 35]. Treatment guidelines for similar features in SLE [29], SSc [27, 28] or RA [31] as well as local drug availability and personal expertise might impact treatment choices in MCTD [2, 35]. In our cohort, the most frequently prescribed agents were hydroxychloroquine, prednisone, methotrexate and rituximab. The fact that continued combination therapy (15/28 patients, 54%) was more frequent than monotherapy (10/28 patients, 36%) and that only few patients (3/28 patients, 11%) remained untreated reflects the severity of this multi-organ disease and ties in with the persistent disease activity that was observed despite immunomodulatory treatment. This observation reflects a large variance in individual outcomes [2, 36–38], including potentially fatal complications such as pulmonary hypertension [37, 38], interstitial lung disease [39] or manifest anti-phospholipid syndrome [38], and contrasts with the initial concept of MCTD as a rather mild disease [1, 14]. Measurement of disease activity using SLEDAI-2 K and EUSTAR-AI further demonstrated a significantly better response to treatment of SLE-like than of SSc-like disease manifestations, which is consistent with previous data [14, 40]. Drug-related adverse events were common and included cytopenia, exanthema, infections and elevated liver enzymes.

The limitations of our study mainly arise from the rather low cohort size. On the other hand, the relatively small number of patients even at a tertiary referral centre also reflects the low disease prevalence and our strict inclusion criteria. The patients of our cohort, however, were well-characterised and prospectively followed with a representative median observation time and minimum observation time of 1 year.

## Data Availability

Data are available upon reasonable request by contacting the corresponding author.
